# The effect of platelet rich plasma combined with celecoxib on knee function and pain in patients with knee osteoarthritis

**DOI:** 10.12669/pjms.38.4.5558

**Published:** 2022

**Authors:** Mingjun Nie, Jianzhong Zhao, Guangcheng Zhang, Jiazhu Tang, Wei Zhu, Qing Zhang

**Affiliations:** 1 Mingjun Nie, Department of Orthopedics, Affiliated Hospital of Jiangsu University, Zhenjiang 212001, Jiangsu Province, P.R. China; 2 Jianzhong Zhao, Department of Orthopedics, Affiliated Hospital of Jiangsu University, Zhenjiang 212001, Jiangsu Province, P.R. China; 3 Guangcheng Zhang, Department of Orthopedics, Affiliated Hospital of Jiangsu University, Zhenjiang 212001, Jiangsu Province, P.R. China; 4 Jiazhu Tang, Department of Orthopedics, Affiliated Hospital of Jiangsu University, Zhenjiang 212001, Jiangsu Province, P.R. China; 5 Wei Zhu, Department of Orthopedics, Affiliated Hospital of Jiangsu University, Zhenjiang 212001, Jiangsu Province, P.R. China; 6 Qing Zhang, Department of Orthopedics, Affiliated Hospital of Jiangsu University, Zhenjiang 212001, Jiangsu Province, P.R. China

**Keywords:** Knee osteoarthritis, Platelet rich plasma, Celecoxib, Knee function, Retrospective analysis

## Abstract

**Objectives:**

To analyze the immediate effect of platelet rich plasma, combined with celecoxib, on knee function and pain in patients with knee osteoarthritis.

**Methods:**

The clinical data of 86 patients with knee osteoarthritis, treated in our hospital from January 2019 to January 2021, were analyzed retrospectively. According to the treatment records, patients were divided into a control group (n = 43, celecoxib) and a treatment group (n = 43, platelet rich plasma + celecoxib). The knee function, pain and clinical effect in the two groups were compared and analyzed using the Hospital for Special Surgery (HSS) knee score and the visual analog scale (VAS).

**Results:**

The treatment group had a higher HSS score, and a lower VAS score compared to the control group (P<0.05). The clinical efficacy in the treatment group was higher than that in the control group (95.35% and 72.09% respectively, P<0.05).

**Conclusions:**

Platelet rich plasma combined with celecoxib can promote the recovery of knee function and reduce pain in patients with knee osteoarthritis. This treatment combination also has a high immediate clinical effectiveness but needs further evaluation to find out the long term effects.

## INTRODUCTION

Knee osteoarthritis is a progressive degeneration of the articular cartilage of the synovial joint which changes the joint edge and cartilage tissue. This leads to hyperplasia of the articular capsule fiber, joint pain, and joint function loss, and severely reduces the quality of daily life of patients.^1^,^2^ Presently, there is a focus on symptomatic support and drug treatment for knee osteoarthritis. Celecoxib is a common therapeutic drug for patients with knee osteoarthritis, which can reduce pain and improve knee function, but may cause adverse reactions, such as nausea, vomiting and dizziness, which limits the scope of its application.

Platelet rich plasma in combination with celecoxib is a new treatment for knee osteoarthritis. This combination may improve the symptoms associated with osteoarthritis and restore the knee function of patients.^3^,^4^ The main goal of this study was to further explore the immediate clinical efficacy of platelet rich plasma in combination with celecoxib on the pain and knee function of patients with osteoarthritis.

## METHODS

The clinical data of 86 patients with knee osteoarthritis treated in our hospital from January 2019 to January 2021 were analyzed retrospectively. According to the treatment records, 43 patients were treated orally with celecoxib and were selected as the control group, and 43 patients were treated with platelet rich plasma + celecoxib and were selected as the treatment group. All patients were treated for three months as described below. The study was approved by the ethics committee (Approval number: KY2021K0802, Date: August 16, 2021), and the patients and their families were informed and cooperated with the study.

### Inclusion criteria:

- Patients diagnosed with knee osteoarthritis by X-ray, MRI and joint fluid examination and classified as grade I ~ III based on Kellgren Lawrence grading system.
- Patients over 50 years of age,
- No previous history of hemorrhagic diseases (such as abnormal coagulation function or Henoch Schonlein purpura).

### Exclusion criteria:

- Patients with systemic infectious diseases and inflammatory arthritis.
- Mental abnormalities or cognitive impairment and cannot cooperate to complete the treatment.
- Poor tolerance to the medication in this study.
- Immune related or gouty arthritis,
- Received anticoagulant therapy one week before enrollment.

The control group was treated with celecoxib provided by Pfizer pharmaceuticals LLC (sub package of Pfizer Pharmaceutical Co., Ltd.), gyzz J: 20120063; Specification: 0.2g; Usage: 0.2g once a day, swallowed with warm boiled water for a total of three months.

The treatment group was treated with platelet rich plasma and celecoxib. The detailed method of the development of the platelet rich plasma was as follows: 5ml whole blood was extracted and centrifuged. The centrifugation speed was 37.5r per minute, the centrifugation time was 15minutes, and the platelet rich plasma was separated from the upper plasma (2ml). The patient was instructed to remain in a supine position with their knee joint flexed. The depression 1cm outside the lower edge of the patella and the patellar ligament was considered the puncture point, and 2ml of the platelet rich plasma was to be slowly injected once a week, three times a month for three months. If the patient had more effusion in the joint cavity, the effusion was extracted before the plasma injection.

Medical records, assessing knee function and pain of the two groups, were compared and analyzed after three months of treatment. Knee function was assessed based on the Hospital Special Surgery knee score (HSS). The scale included six items such as pain, walking function and stability, with a total of 100 points. The higher the score, the better the knee function.^5^ Degree of pain was assessed using the visual analogue scale (VAS) of standard pain, with a total score of 0 ~ 10, with 0 indicating no pain and 10 indicating severe pain. The higher the score, the stronger the pain.^6^ The clinical efficacy of the two groups was observed and the outcomes were assessed using these factors.^7^

After treatment, the symptoms of joint stiffness, redness, swelling and the degree of pain were assessed as measures of the ability of the treatment to cause immediate relief of symptoms. If immediately after the treatment, the patient’s symptoms were significantly improved, and the activity function was not limited, the treatment was considered remarkedly effective. If the patient’s symptoms were partially improved, and the activity function was only partially limited, the treatment was considered remarkedly effective. If after treatment, the patient’s related symptoms did not change and their activity function was limited, the treatment was considered ineffective. The number of effective cases for each group were carefully recorded, and the overall efficacy was calculated: [Effective rate = (effective (control) + effective (treatment) / total].

The data obtained from the study were entered into Excel 2019 for proofreading and processed by SPSS 22.0 software. Measurement data were expressed as mean ± standard deviation (x̄±SD), while the counting data were expressed as a percentage (%). Independent samples t-tests were used for comparison between groups and a paired t-test was used to compare before and after treatment data. Chi square test was used for count data; P<0.05 indicates statistical difference.

## RESULTS

A total of 86 patients with knee osteoarthritis were included in this study, 43 in the control group and 43 in the treatment group. The proportion of men and women in the control group was 25:18, respectively. The age of patients ranged from 50 to 84 years, with an average of (65.47±3.12) years. The duration of illness ranged from three months to four years, with an average of (1.52±0.36) years. The proportion of men and women in the treatment group was 24:19, respectively. The age of patients ranged from 50 to 83 years, with an average of (65.38±3.14) years. The duration of illness ranged from three months to four years, with an average of (1.53 ± 0.35) years. There was no difference in baseline characteristics between the groups (P>0.05).

Knee function and pain after three months of treatment between the two groups is presented in Table-I. Before the treatment, the HSS scores of the treatment and control groups were not different (36.58±3.42 vs. 36.52±3.38). After the treatment, the HSS score in the treatment group was higher than in the control group (72.85±5.63 vs. 65.33±5.29; P<0.05). Before the treatment, the VAS score of the treatment and control groups were similar (8.62±1.24 vs. 8.59±1.22). After the treatment, the VAS score in the treatment group was lower than in the control group (3.26±1.02 vs. 5.87±1.06; P<0.05).

**Table-I:** Comparison of knee function and pain between the two groups (x¯±SD, score).

Group	n	HSS score		VAS score	

		Before treatment	After treatment	Before treatment	After treatment
Treatment group	43	36.58±3.42	72.85±5.63	8.62±1.24	3.26±1.02
Control group	43	36.52±3.38	65.33±5.29	8.59±1.22	5.87±1.06
*t*		0.082	6.383	0.113	11.634
*P*		0.935	0.000	0.910	0.000

The efficacy of the treatment is summarized in Table-II. Of the 43 patients in the treatment group, the remarkable effect of the treatment was reported in 25 patients, treatment was effective in 16 patients, and no improvement was reported in two cases. In contrast, of a total of 43 patients in the control group, 16 patients reported remarkable improvement, good improvement was achieved in 15 cases and no improvement at all in ten cases. The results show that the total efficacy of the treatment group was significantly higher than the control group (95.35% and 72.09% respectively, P<0.05).

**Table-II:** Comparison of clinical efficacy between the two groups [n (%)].

Group	n	Remarkable effect	Effective	Invalid	Total effective rate (%)
Treatment group	43	25	16	2	41 (95.35)
Control group	43	16	15	10	31 (72.09)
*x^2^*					19.848
*P*					0.000

## DISCUSSION

The results from this study showed that platelet rich plasma, in combination with celecoxib for treatment of knee osteoarthritis, can significantly improve knee function, reduce pain, and has a higher clinical effective rate, when compared to treatment with celecoxib alone. This study supports the use of platelet rich plasma as an effective therapeutic option for patients with knee osteoarthritis.

Knee osteoarthritis is a chronic degenerative disease, which is mainly caused by a variety of factors such as the decrease of growth factors in the joint cavity, the increase of inflammatory factors, the disorder of cartilage nutrition and metabolism, excessive weight-bearing, and knee injury, among others. Clinical symptoms, such as knee pain and swelling, seriously reduce patients’ quality of life.^8^ The function of articular cartilage degrades slowly with age. After the structural tissue of the knee joint is damaged or changed, the hyaluronic acid, which is needed for lubrication and protection in the patient’s articular cavity, may accelerate the articular cartilage decomposition. As the population ages, the incidence of knee osteoarthritis has been gradually increasing. At present, the prevalence of knee osteoarthritis in China is over 85%, and its rate increases with age.^9^ If patients with knee osteoarthritis are treated early enough, their knee function may be damaged leading to joint instability. In severe cases, knee varus and valgus deformity may be induced, causing significant pain, and seriously affecting the mobility of patients.^10^

At this stage, osteoarthritis patients are given routine symptomatic support treatment and analgesic treatment to alleviate the symptoms of joint pain, improve their prognosis, and promote recovery. Celecoxib is a commonly prescribed, non-steroidal anti-inflammatory and analgesic medication that can inhibit prostaglandins, reduces the inflammatory response and improves overall function of joints.^11^ However, osteoarthritis patients typically have poor tolerance to medication, reducing the treatment effect and limiting the scope of its clinical application.^12^,^13^ Platelet rich plasma is a new method for clinical treatment of knee osteoarthritis. Platelet rich plasma contains high concentrations of platelets, fibrin and leukocytes.^14^ Leukocytes can prevent infection, reduce inflammatory response, inhibit antagonizing growth factors, and repair diseased tissues. Fibrin can repair local tissue, while platelets can activate growth factors providing nutrients for diseased tissues and repairing joint function.^15^ The combination of platelet rich plasma and celecoxib effectively combines the advantages of the two treatment methods to significantly reduce knee pain and promote a faster recovery of knee function. This study found that the HSS and VAS scores of the treatment group that received a combination of platelet rich plasma and celecoxib, were significantly better than those of the control group (P<0.05). As the source of platelet rich plasma is the patient’s own whole blood, injection into the patient’s joint cavity can activate the immune system, enhance self-healing abilities, accelerate circulation, and enhance the recovery of lesion tissue function. Additionally, platelet rich plasma has a high safety and biocompatibility rating, and does not induce serious adverse reactions in patients, effectively improving the degree of treatment cooperation within the patient population.

We also found that the immediate clinical efficacy in patients, treated with both platelet rich plasma and celecoxib, was significantly higher than that of the control group, treated only with celecoxib (P<0.05). These results suggest that the combination of platelet rich plasma and celecoxib can improve the treatment effect and promote faster recovery. The use of platelet rich plasma can effectively increase the content of platelet rich plasma in a patients’ knee joint through supplementation of exogenous platelet plasma without aggravating local lesions within the knee joint. The combination of platelet rich plasma and celecoxib can further enhance the immediate therapeutic effect, promoting a faster recovery of knee function, and improve the prognosis of patients, with no further increase in adverse reactions. However, further studies, assessing whether the effect of the treatment extends for a long period of time are needed.

### Limitations of the study:

It is a retrospective study with a relatively small sample size of 86 patients. The study duration was limited to three months, and while we were able to discern a significant effect of treatment, we did not follow up the patients for more than one year to understand the effects of this treatment on longer term patient health.

## CONCLUSION

The results presented in this study show that patients with knee osteoarthritis given platelet rich plasma in combination with celecoxib, reported significantly improved knee function and reduced knee pain immediately after three months of treatment. This drug combination also resulted in a significantly better clinical effective rate when compared to the control group, with no further increase in adverse reactions. This study provides evidence of the immediate benefits of combining platelet rich plasma with celecoxib for patients with knee osteoarthritis.

### Authors’ contributions:

**MN** **&** **QZ:** conceived and designed the study.

**JZ, GZ, JT & WZ** collected the data and performed the analysis.

**MN** was involved in the writing of the manuscript and is responsible for the clinical integrity of the study.

All authors have read and approved the final manuscript.
